# Assessment of spatial genetic structure to identify populations at risk for infection of an emerging epizootic disease

**DOI:** 10.1002/ece3.6161

**Published:** 2020-04-22

**Authors:** William L. Miller, Cassandra M. Miller‐Butterworth, Duane R. Diefenbach, W. David Walter

**Affiliations:** ^1^ Pennsylvania Cooperative Fish and Wildlife Research Unit Department of Ecosystem Science and Management Intercollege Graduate Degree Program in Ecology The Pennsylvania State University University Park PA USA; ^2^ Penn State Beaver Monaca PA USA; ^3^ U.S. Geological Survey Pennsylvania Cooperative Fish and Wildlife Research Unit The Pennsylvania State University University Park PA USA

**Keywords:** chronic wasting disease, disease spread, gene flow, hierarchical genetic structure, landscape genetics, *Odocoileus virginianus*, white‐tailed deer

## Abstract

1. Understanding the geographic extent and connectivity of wildlife populations can provide important insights into the management of disease outbreaks but defining patterns of population structure is difficult for widely distributed species. Landscape genetic analyses are powerful methods for identifying cryptic structure and movement patterns that may be associated with spatial epizootic patterns in such cases.
2. We characterized patterns of population substructure and connectivity using microsatellite genotypes from 2,222 white‐tailed deer (*Odocoileus virginianus*) in the Mid‐Atlantic region of the United States, a region where chronic wasting disease was first detected in 2009. The goal of this study was to evaluate the juxtaposition between population structure, landscape features that influence gene flow, and current disease management units.
3. Clustering analyses identified four to five subpopulations in this region, the edges of which corresponded to ecophysiographic provinces. Subpopulations were further partitioned into 11 clusters with subtle (*F*_ST_ ≤ 0.041), but significant genetic differentiation. Genetic differentiation was lower and migration rates were higher among neighboring genetic clusters, indicating an underlying genetic cline. Genetic discontinuities were associated with topographic barriers, however.
4. Resistance surface modeling indicated that gene flow was diffuse in homogenous landscapes, but the direction and extent of gene flow were influenced by forest cover, traffic volume, and elevational relief in subregions heterogeneous for these landscape features. Chronic wasting disease primarily occurred among genetic clusters within a single subpopulation and along corridors of high landscape connectivity.
5. These results may suggest a possible correlation between population substructure, landscape connectivity, and the occurrence of diseases for widespread species. Considering these factors may be useful in delineating effective management units, although only the largest features produced appreciable differences in subpopulation structure. Disease mitigation strategies implemented at the scale of ecophysiographic provinces are likely to be more effective than those implemented at finer scales.

## Graphical abstract

We characterized the spatial population structure of white‐tailed deer in the Mid‐Atlantic region of the United States in order to evaluate the juxtaposition population structure, gene flow patterns, and chronic wasting disease cases in the region. We found evidence of four to five subpopulation units, the extents of which generally corresponded with ecophysiographic provinces, with 11 subtle genetic clusters nested hierarchically within subpopulation units. Landscape genetic analyses revealed corridors of high connectivity adjacent to ridges, roads, and open areas, which may indicate directed disease transmission in regions heterogeneous for these features.

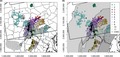

## 1 INTRODUCTION

Emerging wildlife diseases are being increasingly recognized as an important threat to the health of wildlife populations (Sutherland et al., 2018). Many species affected by emerging wildlife diseases have experienced or are predicted to experience population declines or extinctions (Edmunds et al., 2016; Scheele et al., 2019; Thogmartin et al., 2013). Due to the pervasive effects of these diseases, preventing the geographic spread of disease to naïve populations is often a priority of efforts focused on managing emerging wildlife diseases (Langwig et al., 2015). Therefore, understanding the factors that influence the distribution of diseases and predicting patterns of future occurrence have become important objectives for managing diseases. Determining the extent and distribution of wildlife populations can inform disease mitigation strategies because population structure is often correlated with the occurrence and prevalence of wildlife diseases (Blanchong et al., 2008; Cullingham, Kyle, Pond, Rees, & White, 2009).

For species characterized by fidelity to specific habitat patches, the extent of population boundaries and the distribution of associated diseases often correspond to discrete habitat edges. For example, the population structure of little brown bats (*Myotis lucifugus*) and distribution of white‐nose syndrome are closely associated with winter hibernation colonies, movement among which is strongly influenced by local topography (Miller‐Butterworth, Vonhof, Rosenstern, Turner, & Russell, 2014). Many species, however, are habitat generalists with widespread distributions. In cases where populations are continuously distributed on landscapes, spatial population structure often still exists, although subpopulation boundaries can be difficult to delineate (Vergara et al., 2015). A common practice among wildlife specialists is to define population or management units based on geophysical or political boundaries (Rosenberry & Diefenbach, 2019), which may not be reflective of the underlying disease risk for widespread species. Genetic clustering algorithms have become an important tool for defining population substructure and landscape features associated with genetic discontinuities in mobile and widespread species (Coulon et al., 2006). Algorithms that account for spatial autocorrelation of allele frequencies have been shown to be particularly useful for identifying cryptic subpopulation edges in continuously distributed populations (Safner, Miller, McRae, Fortin, & Manel, 2011). For example, Guillot (2008) was able to detect six spatially distinct subpopulations of wolverines (*Gulo gulo*) in a widespread population using a Bayesian clustering algorithm with a correlated allele frequency model where previous efforts based on nonspatial models were able to detect only three (Cegelski, Waits, & Anderson, 2003). By treating allele frequencies as correlated across related clusters as opposed to independently distributed, spatially explicit clustering is more likely to represent the true underlying structure of continuously distributed populations (Guillot, 2008). Thus, clustering methods accounting for genetic autocorrelation are likely to have increased power to detect cryptic subpopulation structure and may outperform other clustering and edge detection methods in identifying features associated with genetic discontinuities (Safner et al., 2011; Vergara et al., 2015). While gene flow may be widespread in continuously distributed populations, disease prevalence rates can vary spatially even with minute deviations from genetic panmixia (Blanchong et al., 2008). Therefore, detection of cryptic genetic discontinuities using spatially explicit clustering methods may provide insights into the potential occurrence and distribution of wildlife diseases affecting widespread species.

The movement of infected individuals among different subpopulations may also influence epizootic patterns at a landscape scale (Green, Manjerovic, Mateus‐Pinilla, & Novakofski, 2014). Dispersal may not occur uniformly and can be influenced by many biological and environmental factors including the permeability of the landscape matrix (Bowler & Benton, 2005). Landscape elements can alter transmission patterns by impeding or directing the movement of infected individuals. For example, the incidence of rabies is concomitant with the permeability of rivers to raccoon (*Procyon lotor*) gene flow, with certain rivers acting as barriers to dispersal and disease transmission (Cullingham et al., 2009). Connectivity analyses have also been demonstrated to be useful for predicting potential transmission corridors based on correlations between gene flow and landscape composition in areas where diseases are recently emergent (Paquette, Talbot, Garant, Mainguy, & Pelletier, 2014). Considering landscape connectivity jointly with patterns of subpopulation structure may improve efforts to mitigate the geographic diffusion of wildlife diseases at a landscape scale by identifying subpopulations that may be at risk for establishment and features that facilitate or impede the dispersal of potentially infected individuals, and by extension, transmission of disease among subpopulations.

### 1.1 Chronic wasting disease

Chronic wasting disease is an emerging and fatal prion disease that affects ecologically and culturally important members of the Cervidae family (Miller & Williams, 2004), including free‐ranging white‐tailed deer (*Odocoileus virginianus*), mule deer (*O. hemionus*), elk (*Cervus canadensis*), and moose (*Alces alces*) populations in North America (Carlson et al., 2018). Of these species, white‐tailed deer are often of particular interest since they are the most common and widely distributed cervid in North America (Heffelfinger, 2011), and because they are the primary vector and species affected in areas where chronic wasting disease is emerging, such as eastern and central North America. No effective treatment currently exists, so management strategies typically include surveillance and targeted herd reductions, with the goal of minimizing the geographic diffusion of the disease (Evans, Schuler, & Walter, 2014; Manjerovic, Green, Mateus‐Pinilla, & Novakofski, 2014). While active management strategies, such as targeted culling efforts, can lead to changes in local prevalence rates (Manjerovic et al., 2014; Mateus‐Pinilla, Weng, Ruiz, Shelton, & Novakofski, 2013), chronic wasting disease has continued to spread geographically at broader scales in most disease foci.

Because chronic wasting disease is spread by direct interactions and indirect contacts through shared environments (Saunders, Bartelt‐Hunt, & Bartz, 2012), population structure may influence the transmission of this disease across affected landscapes. Mismatches between the scale of management efforts, the extent of outbreaks, and subpopulation edges may contribute, in part, to the continued diffusion of this disease. White‐tailed deer also maintain high rates of dispersal (Long, Diefenbach, Rosenberry, Wallingford, & Grund, 2005; Lutz, Diefenbach, & Rosenberry, 2015), so movement is also likely to influence transmission at broader scales in areas where chronic wasting disease is emerging as well. While barriers to movement are permeable, dispersal and gene flow patterns are altered by anthropogenic and topographic barriers (Blanchong et al., 2008; Long, Diefenbach, Wallingford, & Rosenberry, 2010; Lutz, Diefenbach, & Rosenberry, 2016; Robinson, Samuel, Lopez, & Shelton, 2012), which may in turn slow the geographic diffusion of chronic wasting disease (Hefley, Hooten, Russell, Walsh, & Powell, 2017). Evaluating the genetic structure of white‐tailed deer and the extent of connectivity among delineated subpopulations may aid in predicting future epizootic patterns and improve disease mitigation efforts.

### 1.2 Objectives

Here, we have evaluated the spatial population structure and genetic connectivity of white‐tailed deer in a large area of the Mid‐Atlantic region of the United States where chronic wasting disease is an emerging wildlife pathogen. We also assessed the relative resistance of landscape variables hypothesized to affect gene flow in order to identify potential disease transmission corridors. We hypothesized that patterns of population structure would be subtle and gene flow would be widespread. Despite this, we predicted that rivers, topography, the availability of forest cover, and highways with high volume traffic would modulate the extent and directionality of deer gene flow (Blanchong et al., 2008; Kelly et al., 2014; Locher, Scribner, Moore, Murphy, & Kanefsky, 2015; Long et al., 2010; Robinson et al., 2012). We also predicted that subpopulations would be arranged hierarchically because dispersal barriers were expected to be permeable to deer movement.

## 2 MATERIALS AND METHODS

### 2.1 Study area and sample collection

**FIGURE 1:**
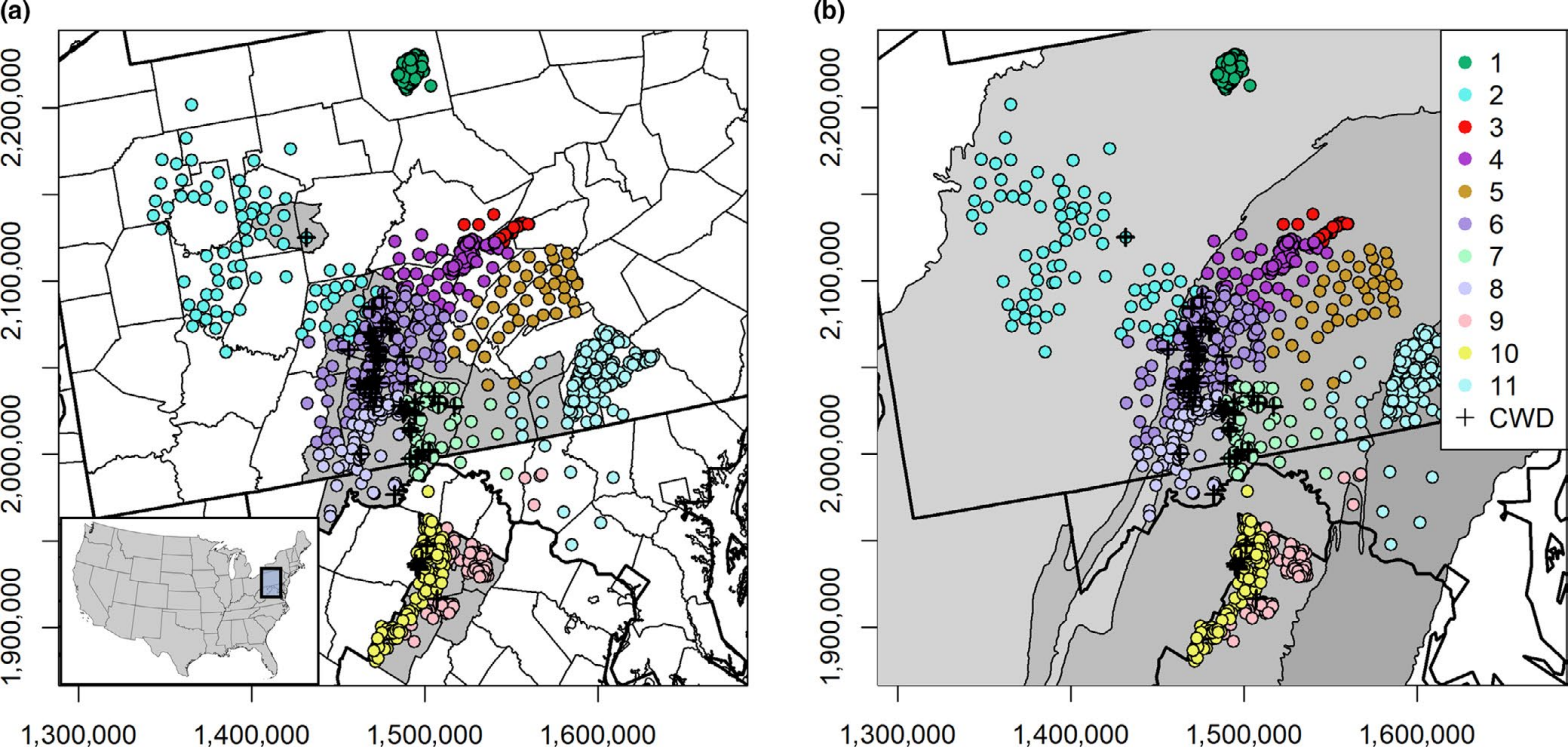
Distribution of 11 white‐tailed deer subpopulations (colored circles) in the Mid‐Atlantic region of the United States. Spatial records for 68 chronic wasting disease (CWD) cases detected from 2009 to 2017 in Pennsylvania, Maryland, and Virginia are displayed as crosses. (a) Comparison of subpopulation distributions to county boundaries with disease management areas highlighted in gray. (b) Comparison of subpopulation distributions to ecophysiographic provinces boundaries (northwest = Allegheny Plateau, central = Ridge‐and‐Valley, southeast = Piedmont)

From 2013 to 2017, we collected tissue samples (*n* = 2,222), consisting primarily of connective or muscle tissue biopsies, from an area encompassing 82,000 km^2^, which included samples from Pennsylvania, Virginia, and Maryland (Figure 1). The sampling region spans three ecophysiographic provinces (Piedmont, Ridge‐and‐Valley, and Appalachian Plateau) that are topographically distinct and separated by major topographic escarpments (Figure 1). This region is heterogeneous for features predicted to influence the genetic structure of white‐tailed deer and patterns of gene flow, including forest cover, topographic complexity, major highways, and rivers (Figure S1). Samples were collected in conjunction with disease surveillance efforts of state agencies and included samples from hunter harvest, vehicle mortality, and targeted removal. Additional samples were collected from captured deer in northern and central Pennsylvania with protocols approved by The Pennsylvania State University (IACUC protocol 47,054). Locations were recorded as either the centroid of the municipal township, hunting management unit, or 2.59 km^2^ sampling grid cell, or as explicit spatial coordinates, depending on the collection method and agency. Both male and female deer were included in the sample (males = 50.7%, females = 47.9%; unknown = 1.4%), since both sexes are capable of dispersing (Long et al., 2005; Lutz et al., 2015). Tissue samples were suspended in 95% ethanol and stored in a −20°C freezer until DNA extraction.

Locality data for chronic wasting disease cases detected from 2009 to 2017 were also recorded for comparison to patterns of population structure and gene flow. Chronic wasting disease was first recorded in the sampling region in northern Virginia in 2009, but cases were found as early as 2005 in an adjacent state (West Virginia). Since then, additional cases have been detected in free‐ranging herds in western Maryland and central Pennsylvania, but region‐wide prevalence rates in free‐ranging populations are still estimated to be low (≤1%; Evans, Kirchgessner, Eyler, Ryan, & Walter, 2016; Evans et al., 2014). Disease management areas were established in response to the detection of chronic wasting disease in free‐ranging populations in these states, and for surveillance in the proximity of infected captive facilities in Pennsylvania (Figure 1a). The edges of disease management areas have been defined using county boundaries (Virginia), previously established wildlife management units (Maryland), or a combination of previous wildlife management units and potential dispersal barriers, such as roads (Pennsylvania). The distribution of chronic wasting disease continues to expand in this region and has recently been detected in areas outside of established disease foci. White‐tailed deer are the only cervid species affected by the disease in the Mid‐Atlantic region, although cases were detected in 2017 in the proximity of an isolated, remnant elk population in central Pennsylvania.

### 2.2 DNA extraction and microsatellite genotyping

We isolated DNA using the animal tissue protocol for the QIAGEN DNeasy blood and tissue extraction kits (QIAGEN). Tissue digestions were incubated for a minimum of 4 hr to ensure samples were completely lysed, and DNA elutions were carried out with a single 150 µl volume of elution buffer to maximize DNA concentration. We quantified the concentration of extracted DNA (ng/µl) using a NanoDrop spectrophotometer (Thermo Fisher Scientific) and diluted samples to approximately 20 ng/µl. All samples were genotyped using 11 microsatellite loci previously shown to be effective for genotyping in this region (Miller, Edson, Pietrandrea, Miller‐Butterworth, & Walter, 2019). The resulting amplicons were analyzed on an Applied Biosystems genetic analyzer (model 3730 XL) at the Penn State Genomics Core Facility. One negative control (deonized H_2_O) was included on each plate in order to ensure amplicons were not contaminated by external sources of DNA and at least one previously genotyped sample was included as well to confirm reproducibility. We used GeneMarker (Softgenetics) to determine allele identity. Alleles were binned using the MsatAllele R package (version 1.5; Alberto, 2009), which can account for imperfect repeat motifs known to impact several of these loci (Miller et al., 2019). We tested for the presence of genotyping errors, null alleles, and deviations from equilibrium assumptions to determine data quality (Appendix S1).

### 2.3 Subpopulation structure

The Bayesian clustering method implemented in the Geneland R package (version 4.6; Guillot, Mortier, & Estoup, 2005) was used to identify the number of genetic clusters (*K*) and delineate population structure. Geneland was chosen as the basis for analysis because it is suggested to be better able to identify cryptic genetic discontinuities than alternative clustering algorithms (e.g., TESS, BAPS) and edge detection methods (e.g., Wombling) in scenarios where gene flow is widespread and dispersal barriers are permeable to movement (Safner et al., 2011), patterns which were predicted of white‐tailed deer in this region based on previous movement studies (Long et al., 2005, 2010; Lutz et al., 2015, 2016). We estimated *K* using the correlated allele frequencies model and a spatial uncertainty term set to 7.071 km, which corresponded to the axial edge of a 50 km^2^ square centered on the sample coordinates. We determined the number of genetic clusters by evaluating 20 levels of *K* using five independent runs with the following parameters: 500,000 Markov Chain Monte Carlo (MCMC) iterations thinning after every 100th iteration, a maximum number of nuclei set to 6,666 (three times the number of samples), and a maximum rate of the Poisson process equal to 2,222 (the number of samples). All postprocess analyses were conducted with a burn‐in period of 1,000 iterations. Cluster assignment was determined using the single run with the highest average log posterior density (Guillot, 2008).

Patterns of genetic diversity were summarized for each inferred genetic cluster using GenAlEx (version 6.5; Peakall & Smouse, 2006, 2012). Specifically, we calculated the average number of alleles per locus (*N*_A_), observed heterozygosity (*H*_O_), unbiased expected heterozygosity (*H*_E_), and the number of private alleles (*P*_A_) for each genetic cluster. Genetic connectivity among inferred genetic clusters was described using migration estimates from BayesAss (version 1.3; Wilson & Rannala, 2003). Migration rates were estimated using the following MCMC parameters: (a) a single chain of 21,000,000 iterations with a burn‐in period of 2,000,000 and thinning every 2,000 steps, (b) a prior on the mixing parameter for allele frequencies of 0.2, (c) a prior on the mixing parameter for inbreeding coefficients of 0.2, and (d) a prior on the mixing parameter for migration rates of 0.05. The chosen prior values produced acceptance rates of proposed changes between 20% and 40%, which is within the suggested guidelines for adequate mixing (Wilson & Rannala, 2003). We ran ten independent replicates and selected the run that minimized the Bayesian deviance criterion for evaluation (Meirmans, 2014).

Pairwise estimates of genetic differentiation among genetic clusters were estimated using Weir and Cockerham’s (1984) *F*_ST_ estimator in FSTAT (version 2.9.3.2; Goudet, 1995). Weir and Cockerham's estimator, hereafter simply referred to as *F*_ST_, provides corrections for multiple loci with more than two alleles each (Weir & Cockerham, 1984), making it an appropriate choice for hypervariable loci like microsatellites. We used 5,500 permutations of the data to calculate *p*‐values. We determined significant deviations from panmixia using a Holm‐Bonferroni procedure to correct for multiple comparisons (Holm, 1979). We created a subpopulation dendrogram to evaluate hierarchical relationships among inferred genetic clusters using the single‐linkage method and pairwise *F*_ST_ values as a measure of genetic distance. An analysis of molecular variance was also used to determine the hierarchical partitioning of genetic variance at three scales: (a) among individuals within genetic clusters, (b) among genetic clusters within larger subpopulation units determined from the hierarchical clustering analysis, and (c) among all subpopulation units. Hierarchical F‐statistics and covariance components were calculated using the poppr R package (version 2.7.1; Kamvar, Tabima, & Grünwald, 2014).

A spatial principal components analysis, carried out in the adegenet R package (version 2.1.1; Jombart, 2008), was used to identify genetic clusters independent of the Geneland analysis for comparison. The connectivity network was defined using a maximum distance of 30 km (“neighborhood by distance option”), which corresponds to a dispersal probability of <5% for white‐tailed deer in this region (Diefenbach, Long, Rosenberry, Wallingford, & Smith, 2008). The number of clusters and cluster membership was identified using two methods. We plotted the lag scores for the first two principal components to visually represent the distribution of local genetic variability. *K*‐means clustering was also used to determine the most likely number of clusters based on lag scores. The optimum number of genetic partitions was chosen using the “elbow method” (Ketchen & Shook, 1996).

### 2.4 Landscape genetics

We evaluated the relationship between genetic connectivity within and among genetic clusters using a resistance surface modeling approach in order to identify potential corridors of movement and disease transmission. Samples were regrouped using a 25 × 25 km sampling grid matched to the extent of each identified genetic cluster in order to maintain a more equal sampling distribution conducive to landscape genetic analyses, while at the same time preserving cluster assignments. While two genetic clustering methods were used, we chose to use the method that produced the finest‐scale genetic partitions for landscape genetic analyses since these edges would be more reflective of subtle landscape barriers. Any grid that incorporated <20 samples was excluded from further analyses. Pairwise *F*_ST_ values were estimated among subsampling units following the same methods described previously.

Rasters representing landscape variables predicted to influence gene flow were created and processed in ArcGIS Desktop 10.5.1 (ArcMap; Environmental Systems Research Institute; Figure S1). Continuous features included forest cover (percent per grid cell; created using the National Land Cover Database 2011; Homer et al., 2015), elevational relief (range of elevation values in a 3 × 3 pixel neighborhood; created using the National Elevation Dataset, courtesy of the U.S. Geological Survey), a proxy for topographic complexity, and traffic volume (average annual daily traffic; created using the 2015 Highway Performance Monitoring System dataset, courtesy of the Federal Highway Administration), a metric used to represent road size and use. Large streams (Strahler order ≥4) were represented with a binomial raster (created using the 2012 National Atlas of the United States of America, courtesy of the U.S. Geological Survey). Rasters were scaled to a pixel size of 6 km^2^, which corresponds to the average home range size of white‐tailed deer in this region (Evans et al., 2014).

Resistance values were chosen concurrently for all rasters using a genetic optimization algorithm implemented in the ResistanceGA R package (version 4.0‐5; Peterman, 2018). A mutation rate of 0.125 and a crossover rate of 0.850 were used to generate resistance values for each iteration. Deer gene flow was modeled using random‐walk commute distances for each realization of the resistance surface. We used the log‐likelihood of a maximum‐likelihood population effects model to evaluate the correlation between genetic distance and resistance distance at each iteration as the selection rule. The exploration operators included a maximum of 1,000 iterations, a convergence rule of termination after 50 iterations without improvement, and a joint maximum resistance of 5,000 per pixel. Since the optimization algorithm is a stochastic process, we produced five replicate runs of the optimization procedure and evaluated the model with the highest log‐likelihood. Because white‐tailed deer population structure is predicted to display an underlying pattern of isolation‐by‐distance (Kelly et al., 2014; Locher et al., 2015; Robinson et al., 2012), we also evaluated a distance‐only model for comparison. Models were ranked based on Akaike's information criterion corrected for small sample size (AICc). Current density maps were also produced to display connectivity corridors using CIRCUITSCAPE (version 4.0; McRae, Dickson, Keitt, & Shah, 2008).

## 3 RESULTS

### 3.1 Population structure

The Geneland algorithm identified 10–13 subpopulations across five independent runs. The value of *K* for the iteration with the highest log posterior density was 13 (log posterior density = −84,763.034); however, the MCMC output indicated poor convergence relative to the chain with the second‐best run (Appendix S2). Thus, we elected to evaluate spatial genetic patterns based on the chain with the second highest log posterior density (*K* = 11; log posterior density = −85,614.685; Figure 1), which had parsimonious genetic partitions and exhibited model convergence. These subpopulations were characterized by high allelic diversity (average *N*_A_ = 13.47) and heterozygosity (mean *H*_O_ = 0.801; *H*_E_ = 0.841) but a low number of private alleles (mean *P*_A_ = 0.727; Table 1). Genetic differentiation among genetic clusters was low (average *F*_ST_ = 0.019; range = 0.004–0.041; Table S1), although significant in all pairwise comparisons (*p* < .0009). Average immigration rates among genetic clusters were high (*m*_imm_ = 0.171; range = 0.035–0.326; Table 1; Table S2). The proportion of resident individuals approached the lower bounds for this parameter for three populations (<0.73; Meirmans, 2014). High immigration rates from neighboring genetic clusters were causative in all cases (Table S2). Thus, migration rates were interpreted as relative measures of genetic connectivity rather than absolute estimates.

**TABLE 1:** Genetic summary statistics and sample sizes (*N*) for 11 white‐tailed deer genetic clusters in the Mid‐Atlantic region of the United States inferred from Geneland. Genetic cluster designations correspond to Figure 1. Measures of genetic diversity include allelic richness (*N*_A_), observed heterozygosity (*H*_O_), expected heterozygosity (*H*_E_), and the number of private alleles (*P*_A_). Recent immigration rates (*m*_imm_) were used to define general patterns of genetic connectivity

Cluster	*N*	*N*_A_	*H*_O_	*H*_E_	*P*_A_	*m*_imm_
1	324	14.000 (1.136)	0.820 (0.028)	0.850 (0.027)	1	0.064
2	245	13.818 (1.007)	0.800 (0.033)	0.842 (0.032)	1	0.059
3	46	11.545 (0.743)	0.812 (0.026)	0.851 (0.024)	1	0.326
4	269	13.364 (0.937)	0.784 (0.024)	0.829 (0.028)	0	0.144
5	94	13.091 (1.022)	0.805 (0.035)	0.844 (0.030)	0	0.294
6	382	13.727 (1.054)	0.795 (0.034)	0.838 (0.034)	0	0.162
7	154	13.636 (1.089)	0.802 (0.030)	0.841 (0.031)	0	0.137
8	180	14.000 (1.079)	0.804 (0.034)	0.843 (0.036)	0	0.320
9	134	13.727 (1.054)	0.810 (0.024)	0.854 (0.020)	1	0.217
10	159	14.091 (0.967)	0.797 (0.033)	0.825 (0.033)	2	0.120
11	235	13.812 (1.166)	0.783 (0.025)	0.838 (0.021)	2	0.035
*M*	202 (28.744)	13.471 (0.304)	0.801 (0.009)	0.841 (0.008)	0.727 (0.226)	0.171 (0.030)

Note

Values in parentheses represent one standard error for mean estimates.

**FIGURE 2:**
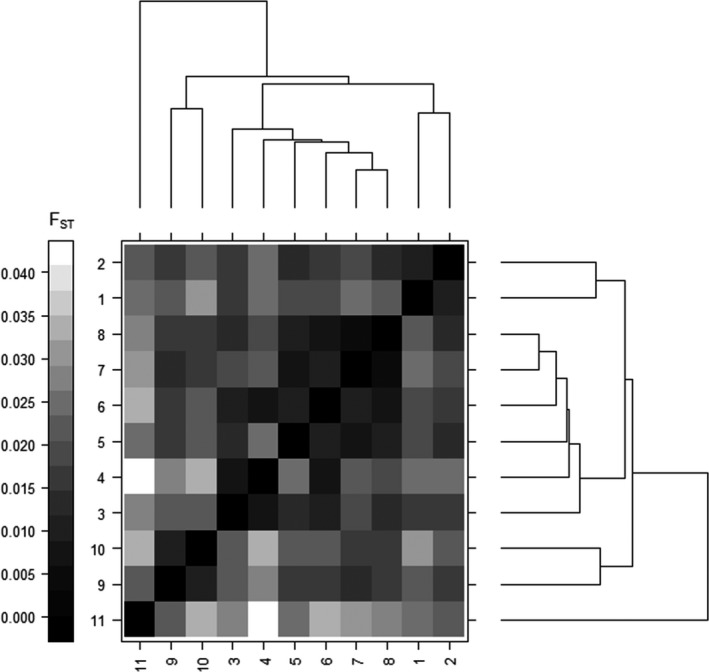
Hierarchical clustering of inferred white‐tailed deer subpopulations in the Mid‐Atlantic region of the United States. The length of each branch corresponds to the degree of genetic divergence (*F*_ST_) among subpopulations

**FIGURE 3:**
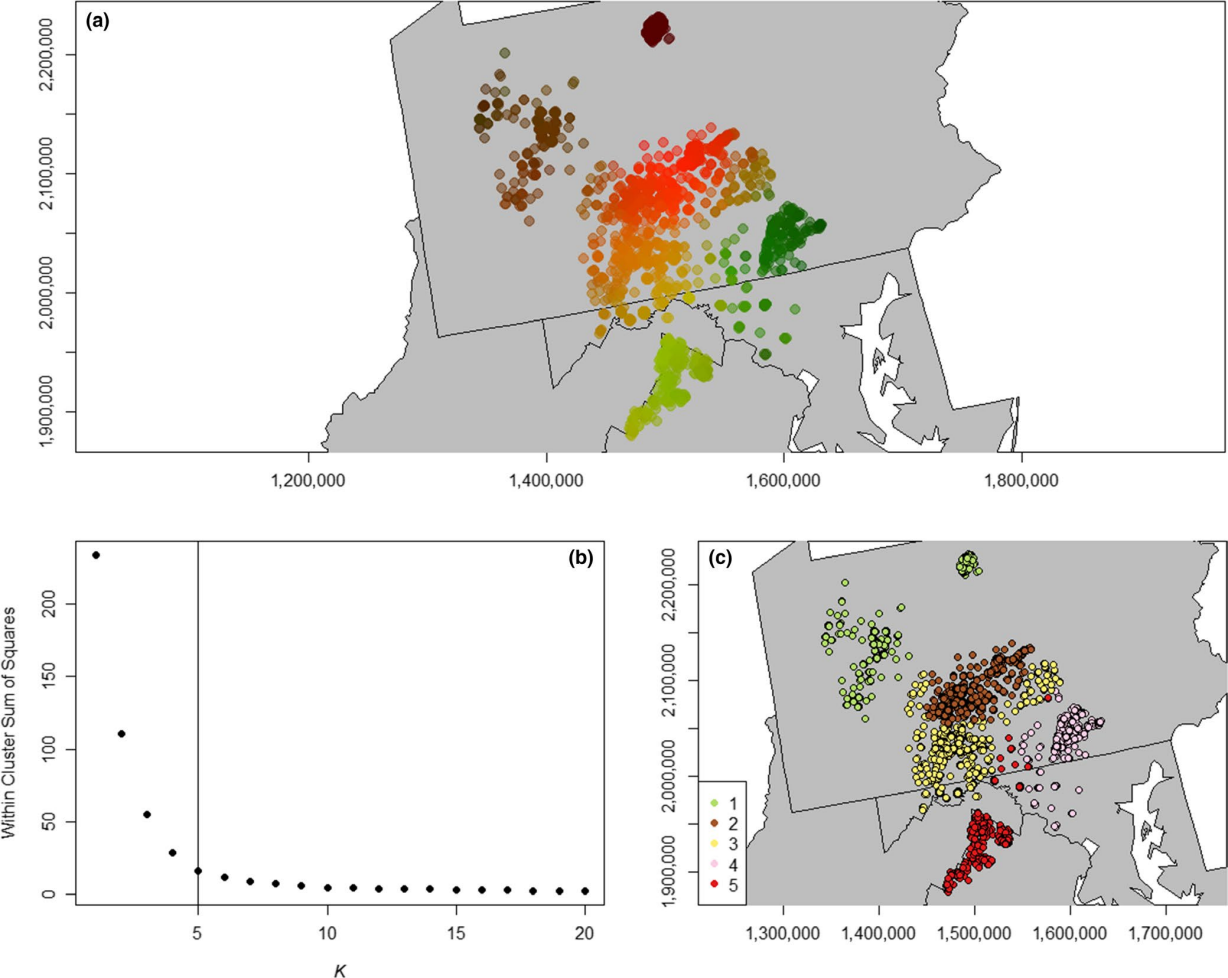
Results of the spatial principal component analysis used for white‐tailed deer from the Mid‐Atlantic region of the United States. (a) Plot of raw lag scores from first and second principal components. (b) Results of *K*‐means clustering of lag scores used to identify the most likely number of clusters (*K* = 5) via the elbow method. (c) Map of five identified clusters

Genetic clusters exhibited a nested pattern using a hierarchical clustering analysis based on genetic distance among inferred clusters. Four hierarchical groups were identified (hereafter referred to as subpopulations, Figure 2). Demarcation of these larger subpopulations generally coincided with the boundaries of ecophysiographic provinces. Inferred migration and gene flow were greatest among genetic clusters in the central Ridge‐and‐Valley population (Table S2). While genetic differentiation was significant among all genetic clusters identified by Geneland within hierarchical subpopulation units (genetic variation explained = 2.38%; *F*_SP_ = 0.024; *p* = .001) and among all subpopulations within the region (genetic variation explained = 1.99%; *F*_PT_ = 0.020; *p* = .001), the greatest amount of genetic variation was explained by comparisons made among individuals within genetic clusters (genetic variation explained = 95.6%, *F*_IS_ = 0.044; *p* = .001). Visual inspection of the principal components plot and *K*‐means clustering of lag scores identified five genetic clusters that generally corresponded to subpopulation groupings identified in the hierarchical clustering analysis, although the central Ridge‐and‐Valley population was split into two clusters (Figure 3a,c).

### 3.2 Landscape genetics

**FIGURE 4:**
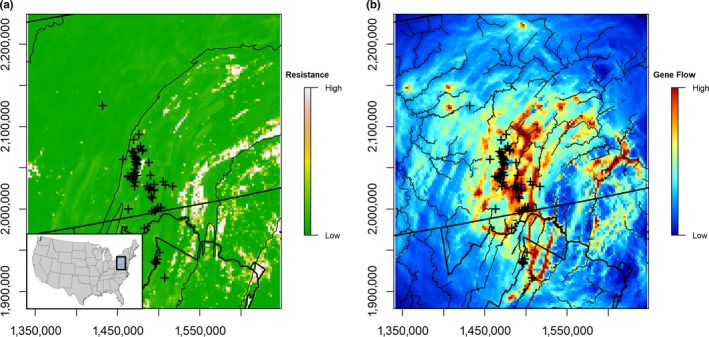
(a) A composite landscape resistance surface for white‐tailed deer in the Mid‐Atlantic region of the United States. Ecophysiographic provinces are outlined. (b) A current density surfaces plot showing patterns of gene flow among subpopulations with streams added to highlight the relationship between riparian corridors and gene flow pathways. Spatial records for 68 chronic wasting disease cases detected from 2009 to 2017 in Pennsylvania, Maryland, and Virginia are displayed as crosses

**FIGURE 5:**
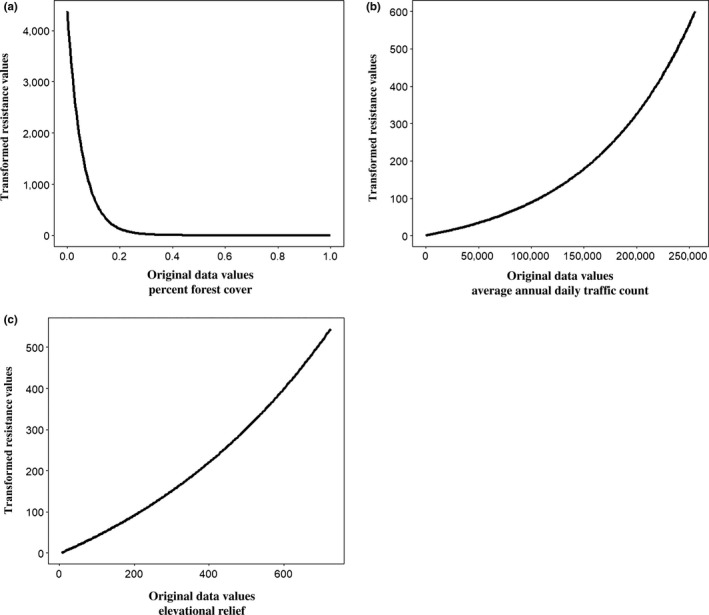
Relationship between pixel values of the raw landscape covariate rasters and the optimized resistance values included in the composite resistance raster used to infer patterns of gene flow for white‐tailed deer. (a) forest cover, (b) highway traffic volume, and (c) topographic heterogeneity

Subpartitioning each of the 11 genetic clusters using a 25 × 25 km sampling grid resulted in 34 sampling units for landscape genetics analyses with sample sizes ranging from 20 to 324 (total sample size = 1,796; Figure S1). The Geneland clusters were chosen as a basis for this analysis because they represent the finest‐scale genetic partitions identified in the clustering analyses but are also hierarchically nested in broader subpopulation groups. Gene flow among sampling units was most associated with the landscape resistance model (parameters = 9, *β* = 0.016, marginal *R* ^2^ = .664, AICc = −3,953.008) relative to both the isolation‐by‐distance‐only model (parameters = 2, *β* = 0.008, marginal *R* ^2^ = .470, AICc = −3,882.790) and intercept‐only model (parameters = 1, *β* = 0.022, marginal *R* ^2^ = .000, AICc = −3,585.570). Landscape resistance was not spatially uniform and was focused in the southeastern portion of the study domain (Figure 4a). Areas of low forest cover (≤0.20) were the most resistant feature (maximum resistance = 4,373.130). Resistance decreased asymptotically, with percent forest cover values between 0.20 and 1.00 taking similar resistance values (Figure 5a). Traffic volume and elevational relief were also resistant to gene flow, although the effects of each were less than forest cover (maximum resistance = 600.111 and 544.692, respectively). Resistance increased linearly for both features (Figure 5b,c). Large streams did not affect resistance (maximum resistance = 1.971). Current density, which indicates directed gene flow, was higher adjacent to areas of greater elevational relief and roads with high traffic volume, and in interspersed segments of high and low forest cover (Figure 4b). Gene flow was more diffuse in areas of lower elevational relief and more contiguously forested areas (Figure 4b). Corridors for gene flow generally approximated the flow paths of lower Strahler order streams in this region (Figure 4b).

### 3.3 Chronic wasting disease

As of 2017, chronic wasting disease management areas encompassed five of the eight Ridge‐and‐Valley genetic clusters identified in Geneland analyses (Figure 1). In the Ridge‐and‐Valley ecophysiographic province, three genetic clusters were not included in the central disease management area in Pennsylvania (clusters 3.4, and 5) and part of clusters 7 and 9 were not included in the disease management unit in Maryland (Figure 1). The northwestern disease management unit in Pennsylvania overlapped a small portion of a single genetic cluster (Figure 1). Clusters 9 and 10 generally approximated the disease management area of Virginia, although it is difficult to evaluate whether the boundaries of the Virginia disease management area fully encompass these genetic clusters since these also correspond with the southern extent of the sampling region (Figure 1).

We were able to obtain spatial locations for 68 deer positive for chronic wasting disease in the study region. Although we did not have a complete count of all infected deer from 2009 to 2017 in the region, because the disease status of some samples was pending and records were unavailable from neighboring states, the spatial distribution of these cases was representative of epizootic trends in this region during this timeframe. All but two of the 68 records were from the Ridge‐and‐Valley ecophysiographic province, but only one did not cluster with the Ridge‐and‐Valley populations (Figures 1 and 4). Forty‐five deer positive for chronic wasting disease occurred within areas corresponding to the highest predicted probability of deer movement (95th to 100th current density quantiles; Figure 4b).

## 4 DISCUSSION

### 4.1 Population structure and connectivity

White‐tailed deer are common and widespread in the Mid‐Atlantic region, but subtle patterns of population substructure were observed. Our results suggest the presence of four to five subpopulations, the extents of which were consistent with ecophysiographic provinces in this region. Ecophysiographic provinces are defined by large geophysical escarpments in the Mid‐Atlantic region. Geneland also identified fine‐scale discontinuities, the majority of which (8 out of 11) occurred within the topographically heterogeneous Ridge‐and‐Valley province. Ridges can affect the extent and direction of deer movement in this region (Long et al., 2010) and are likely contributing to the formation of subtle genetic discontinuities and subpopulation structure. Deer located in more topographically homogenous regions show no such relationship between ecophysiographic province and population structure (Robinson et al., 2012), where subpopulation edges were more closely associated with anthropogenic barriers, habitat configuration, and rivers (Blanchong et al., 2008; Kelly et al., 2014; Locher et al., 2015; Robinson et al., 2012).

Deer maintained high rates of connectivity within and among genetic clusters throughout the region. Migration rates were high and pairwise estimates of genetic differentiation were low, particularly among proximal clusters. Landscape genetic analyses also indicated a possible underlying genetic cline, with the isolation‐by‐distance model explaining 47% of the variance in pairwise measures of genetic differentiation. This pattern of isolation‐by‐distance is a common characteristic of widespread species (Pelletier et al., 2012; Vergara et al., 2015) and has been documented in other white‐tailed deer populations (Kelly et al., 2014; Locher et al., 2015; Robinson et al., 2012). An isolation‐by‐distance pattern of population structure may also lead to over‐estimating the number of true genetic partitions when using Geneland and other clustering analyses (Frantz, Cellina, Krier, Schley, & Burke, 2009; Safner et al., 2011). The occurrence of genetic discontinuities identified by Geneland and extent of subpopulation structure identified in hierarchical‐ and ordination‐based clustering analyses is likely indicative of true biological trends, however, for several reasons. First, genetic discontinuities and subpopulation edges coincide with barriers expected to affect dispersal in this region, particularly ridges (Long et al., 2010). All pairwise and hierarchical measures of population differentiation also indicated significant deviations from panmixia at all scales. Congruency between the results of the hierarchical clustering and ordination analyses provided further support for the identified subpopulation clusters at broader scales. Finally, despite evidence for an underlying genetic cline, the landscape resistance model was chosen as the best explanatory model. This further indicates that while gene flow may be widespread, the extent and directionality of genetic connectivity were also influenced by the characteristics and composition of the underlying landscape in addition to distance alone in this region (Figure 4a).

Features included in the landscape resistance model, including forest cover, topographical complexity, and roads with high traffic volume, have been previously demonstrated to influence the individual movement patterns of white‐tailed deer in this region (Long et al., 2005, 2010; Lutz et al., 2016). Our results further demonstrate that these landscape features also affect broader patterns of population connectivity. Despite the relationship between genetic discontinuities and topography, areas with <20% forest cover were the most resistant to white‐tailed deer gene flow. Landscape resistance associated with reduced forest cover was highest in the southeastern portion of the study domain, an area with higher human densities and/or more widespread agricultural production relative to other areas. While less resistant to gene flow, high volume traffic roads were also concentrated in these same areas. Human development, agriculture, and roads have all been shown previously to reduce gene flow in anthropogenically modified landscapes (Blanchong, Sorin, & Scribner, 2013; Kelly et al., 2014; Locher et al., 2015; Robinson et al., 2012).Therefore, it is likely that human densities and anthropogenic development (e.g., impervious surface, highways) are combining to reduce deer movement across these open areas in an otherwise predominately forested landscape. The effects of topography on landscape resistance were also less than forest cover but more widely distributed across the region (Figure S1). Therefore, topography is likely having broader effects on regional subpopulation structure, while human barriers have more intense but localized effects.

Features contributing to landscape resistance were responsible for directing patterns of gene flow (Figure 4b). Areas with the highest probability of deer gene flow occurred along forested corridors adjacent to areas of greater elevational relief, roads with high traffic volumes, and/or open areas. Previous studies also demonstrate that landscape barriers change the direction of individual deer movement (Long et al., 2010; Lutz et al., 2016), and our results highlight that landscape barriers can affect the directionality of regional connectivity patterns. Although large streams had very low resistance values, gene flow pathways occurred proximally to streams. Streams are often surrounded by forested riparian buffers that white‐tailed deer are known to follow when dispersing (Walter et al., 2011). Riparian forest cover likely provides pathways through otherwise resistant landscapes composed of agriculture and human development, thus influencing gene flow. Rivers may also provide areas of lower topographic relief that deer can use to cross ridges. Gene flow was less directed in the more homogeneously forested Allegheny Plateau province, possibly indicating that gene flow is largely diffuse as previously reported with dispersal patterns in this region (Long et al., 2010).

While population connectivity was influenced by landscape features, genetic differentiation was low at all hierarchical scales. Our findings are commensurate with prior predictions that barriers are permeable to deer movement (Blanchong et al., 2008; Locher et al., 2015; Long et al., 2010; Robinson et al., 2012). While deer commonly terminate dispersal near topographic and anthropogenic barriers, radiomarked deer have been documented crossing them (Long et al., 2010; Lutz et al., 2016). Therefore, our results support the hypothesis and expand on the scale of assessment that landscape features are not barriers to movement but decrease dispersal to produce subtle changes to populations structure.

### 4.2 Chronic wasting disease management

Chronic wasting disease management zones are often defined by a combination of previously defined wildlife management units, political boundaries, and/or inferred dispersal barriers. Since the diffusion of chronic wasting disease can be facilitated by animal movement (Green et al., 2014), understanding of population substructure and dispersal behaviors may improve disease mitigation strategies when compared to undirected management efforts (Blanchong et al., 2008). Even those strategies that incorporate landscape features associated with potential population discontinuities may be ineffective if the permeability of such barriers is not addressed. For example, a common practice of regional disease management strategies is to define surveillance areas in part by state roads, which had little influence on subpopulation structure and gene flow here and in a previous study (Robinson et al., 2012).

Defining future disease management efforts by features associated with genetic discontinuities at a regional scale may be more effective. In the Mid‐Atlantic region, this corresponds to major geophysical escarpments concomitant with ecophysiographic boundaries and the extent of hierarchical subpopulation units and large, deforested areas associated with human development. Based on our results, genetic clusters or subpopulation units that are separated by such features in this region are hypothesized to be less at risk for dispersal‐mediated disease transmission relative to those that maintain higher rates of connectivity. Differences in landscape features associated with subpopulation structure described here when compared to other studies suggest that landscape‐scale correlations are likely to be context specific, however (Blanchong et al., 2008; Kelly et al., 2014; Robinson et al., 2012). Therefore, genetic monitoring in areas with novel outbreaks may improve management efforts, particularly in areas with a unique landscape context relative to previous studies. Additionally, extrapolation of trends from previous studies may not be as effective a strategy in cases where landscape characteristics differ among regions or where movement patterns differ among populations or species.

Our results may indicate that the occurrence of chronic wasting disease in the Mid‐Atlantic region may be correlated with broader patterns of subpopulation structure. Most cases were concentrated within the Ridge‐and‐Valley subpopulations. Disease management areas partially underapproximated the extent of the subpopulations in this province, however (Figure 1a). Given the high rates of genetic connectivity among Ridge‐and‐Valley genetic clusters, and a pattern of gene flow following a southwest‐to‐northeast axial direction in this province (Figure 4b), we predicted that areas encompassing genetic clusters 3, 4, and 5 may be more likely to be affected by dispersal‐mediated transmission than other regions. In 2019, the extent of the disease management area in central Pennsylvania was expanded to include areas encompassing genetic clusters 3, 4, and 5 due to the detection of novel cases of chronic wasting disease. High rates of connectivity with clusters 6, 7, and 8 may indicate that dispersal‐mediated transmission from areas with established outbreaks remains a plausible scenario of disease establishment. Maryland has also expanded their disease management area eastward to include genetic cluster 7, a genetic cluster where cases were observed in Pennsylvania during the time period of this study. Given the extent of genetic cluster 7, detection of chronic wasting disease in this area is not surprising and highlights the importance of interstate coordination and consistency in disease management efforts since genetic clusters with known disease incidence cross‐state boundaries (Figure 1).

Based on our results, we predict that landscape features associated with broader patterns of subpopulation structure may still influence the distribution of chronic wasting disease in this region. Despite ongoing surveillance in subpopulation clusters neighboring the Ridge‐and‐Valley province, neighboring clusters have either low rates of occurrence (west), or no known occurrence in free‐ranging populations (east). Because of the juxtaposition between hierarchical population structure and ecophysiographic provinces, surveillance and management interventions may be most effective if focused within ecophysiographic provinces with known disease occurrence. This may lead to a greater probability of detecting novel disease occurrences relative to random or spatially uniform sampling strategies implemented at broader or localized scales. Outbreaks in distinct genetic clusters may also have different origins and epizootiological patterns. For example, novel cases of chronic wasting disease in the Allegheny Plateau region had a higher probability of assignment and ancestry to captive deer populations than deer from other ecophysiographic provinces (Miller & Walter, In press). Therefore, disease establishment and transmission may be affected by factors other than connectivity with infected subpopulations, such as egression from captive facilities. Individual‐based analyses focused on assignment of deer infected with chronic wasting disease to the subpopulation clusters described here may further elucidate the relative influence of population substructure, dispersal, and alternative sources of infection, such as captive farms, on the epizootiology of chronic wasting disease in this region.

While defining population substructure can aid in defining the extent of disease management efforts, landscape connectivity models may help to identify potential transmission corridors between infected and naïve groups (Paquette et al., 2014). Many deer with chronic wasting disease occurred within corridors with high predicted probability of movement (Figure 4b). This could suggest that landscape characteristics dictating deer movement may be correlated with the transmission and occurrence of chronic wasting disease in this region. Therefore, landscape resistance models may improve efforts to forecast the continued spread of chronic wasting disease in this and other regions. Gene flow occurred along pathways paralleling resistant features in heterogeneous landscapes in the Mid‐Atlantic region. We predict that chronic wasting disease may be more likely to spread along these connectivity corridors, given previous relationships between white‐tailed deer movement and chronic wasting disease spread (Green et al., 2014). Disease diffusion models also suggest the axial spread of chronic wasting disease along landscape features resistant to deer movement in other regions (Hefley et al., 2017). The proximity of connectivity corridors to forested riparian cover also supports previous predictions that these features are likely to play an important role in deer movement and may affect transmission dynamics of chronic wasting disease in heterogeneous environments (Nobert, Merrill, Pybus, Bollinger, & Hwang, 2016; Walter et al., 2011). Based on our results, we also predict that disease transmission may be more diffusive in homogeneous landscapes, such as those in the Allegheny Plateau, due to the undirected nature of deer movement.

### 4.3 Future directions

Our results help to elucidate patterns of spatial substructure and genetic connectivity in a continuously distributed population of white‐tailed deer where chronic wasting disease is emerging. Population‐level trends may help to describe the relative risk of transmission and provide important insights into future epizootic patterns. Genetic samples from deer with chronic wasting disease were limited in this current study, however, and we did not have access to spatial records of chronic wasting disease from neighboring states with active infection, such as West Virginia. Continued genetic sampling and disease surveillance will clarify genetic cluster membership in areas where samples were currently unavailable and improve efforts to assess the association between subpopulation structure and chronic wasting disease occurrence. Integrating measures of population substructure and connectivity into diffusion models, such as those used in Hefley et al. (2017) to predict the temporal spread of chronic wasting disease, represent a critical extension of the current study for validating the inferred correlation between genetic connectivity, landscape resistance, and transmission risk. Diffusion models will also allow for the quantitative assessment of chronic wasting disease transmission at a landscape scale.

Individual‐based genetic analyses, such as ancestry and assignment analyses, will also allow for testing specific hypotheses regarding the origin of chronic wasting disease cases. For example, Miller and Walter (In press) used simulated reference clusters based on empirical genotypes from wild and captive deer in order to evaluate the influence of captive egression on chronic wasting disease occurrence in free‐ranging populations. Similar analyses would likely be useful in further determining the role dispersal plays in patterns of chronic wasting disease epizootiology. Describing the number of genetic clusters and degree of population connectivity was an important first step in optimizing individual‐based admixture and assignment analyses.

## CONFLICT OF INTEREST

None declared.

## AUTHORS' CONTRIBUTIONS

WLM, DRD, and WDW collected genetic samples. WLM, CMM‐B, and WDW performed microsatellite genotyping. WLW and WDW analyzed the genetic and spatial data. All authors contributed to the preparation of this manuscript.

## Supporting information

Figure S1

Table S1

Table S2

Appendix S1

Appendix S2

## Data Availability

Microsatellite genotypes, locality information, and raster data will be available on Zenodo following a 1‐year embargo period from the date of publication (https://doi.org/10.5281/zenodo.3675373).

## References

[ece36161-bib-0001] Alberto, F. (2009). MsatAllele_1.0: An R package to visualize the binning of microsatellite alleles. Journal of Heredity, 100(3), 394–397. 10.1093/jhered/esn110 19126639

[ece36161-bib-0002] Blanchong, J. A. , Samuel, M. D. , Scribner, K. T. , Weckworth, B. V. , Langenberg, J. A. , & Filcek, K. B. (2008). Landscape genetics and the spatial distribution of chronic wasting disease. Biology Letters, 4(1), 130–133. 10.1098/rsbl.2007.0523 18077240 PMC2412942

[ece36161-bib-0003] Blanchong, J. A. , Sorin, A. B. , & Scribner, K. T. (2013). Genetic diversity and population structure in urban white‐tailed deer. The Journal of Wildlife Management, 77(4), 855–862. 10.1002/jwmg.521

[ece36161-bib-0004] Bowler, D. E. , & Benton, T. G. (2005). Causes and consequences of animal dispersal strategies: Relating individual behaviour to spatial dynamics. Biological Reviews of the Cambridge Philosophical Society, 80(2), 205–225. 10.1017/S1464793104006645 15921049

[ece36161-bib-0005] Carlson, C. M. , Hopkins, M. C. , Nguyen, N. T. , Richards, B. J. , Walsh, D. P. , & Walter, W. D. (2018). Chronic wasting disease—Status, science, and management support by the U.S. Geological Survey. U.S. Geological Survey Open File Report No. 2017–1138; p. 8.

[ece36161-bib-0006] Cegelski, C. C. , Waits, L. P. , & Anderson, N. J. (2003). Assessing population structure and gene flow in Montana wolverines (Gulo gulo) using assignment‐based approaches. Molecular Ecology, 12(11), 2907–2918. 10.1046/j.1365-294X.2003.01969.x 14629372

[ece36161-bib-0007] Coulon, A. , Guillot, G. , Cosson, J.‐F. , Angibault, J. M. A. , Aulagnier, S. , Cargnelutti, B. , … Hewison, A. J. M. (2006). Genetic structure is influenced by landscape features: Empirical evidence from a roe deer population. Molecular Ecology, 15(6), 1669–1679. 10.1111/j.1365-294X.2006.02861.x 16629819

[ece36161-bib-0008] Cullingham, C. I. , Kyle, C. J. , Pond, B. A. , Rees, E. E. , & White, B. N. (2009). Differential permeability of rivers to raccoon gene flow corresponds to rabies incidence in Ontario, Canada. Molecular Ecology, 18(1), 43–53. 10.1111/j.1365-294X.2008.03989.x 19140963

[ece36161-bib-0009] Diefenbach, D. R. , Long, E. S. , Rosenberry, C. S. , Wallingford, B. D. , & Smith, D. R. (2008). Modeling distribution of dispersal distances in male white‐tailed deer. Journal of Wildlife Management, 72(6), 1296–1303. 10.2193/2007-436

[ece36161-bib-0010] Edmunds, D. R. , Kauffman, M. J. , Schumaker, B. A. , Lindzey, F. G. , Cook, W. E. , Kreeger, T. J. , … Cornish, T. E. (2016). Chronic wasting disease drives population decline of white‐tailed deer. PLoS ONE, 11, e0161127 10.1371/journal.pone.0161127 27575545 PMC5004924

[ece36161-bib-0011] Evans, T. S. , Kirchgessner, M. S. , Eyler, B. , Ryan, C. W. , & Walter, W. D. (2016). Habitat influences distribution of chronic wasting disease in white‐tailed deer. The Journal of Wildlife Management, 80(2), 284–291. 10.1002/jwmg.1004

[ece36161-bib-0012] Evans, T. S. , Schuler, K. L. , & Walter, W. D. (2014). Surveillance and monitoring of white‐tailed deer for chronic wasting disease in the northeastern United States. Journal of Fish and Wildlife Management, 5(2), 387–393. 10.3996/032014-JFWM-021

[ece36161-bib-0013] Frantz, A. C. , Cellina, S. , Krier, A. , Schley, L. , & Burke, T. (2009). Using spatial Bayesian methods to determine the genetic structure of a continuously distributed population: Clusters or isolation by distance? Journal of Applied Ecology, 46(2), 493–505. 10.1111/j.1365-2664.2008.01606.x

[ece36161-bib-0014] Goudet, J. (1995). FSTAT (Version 1.2): A computer program to calculate F‐statistics. Journal of Heredity, 86(6), 485–486. 10.1093/oxfordjournals.jhered.a111627

[ece36161-bib-0015] Green, M. L. , Manjerovic, M. B. , Mateus‐Pinilla, N. , & Novakofski, J. (2014). Genetic assignment tests reveal dispersal of white‐tailed deer: Implications for chronic wasting disease. Journal of Mammalogy, 95(3), 646–654. 10.1644/13-MAMM-A-167

[ece36161-bib-0016] Guillot, G. (2008). Inference of structure in subdivided populations at low levels of genetic differentiation—The correlated allele frequencies model revisited. Bioinformatics, 24(19), 2222–2228. 10.1093/bioinformatics/btn419 18710873

[ece36161-bib-0017] Guillot, G. , Mortier, F. , & Estoup, A. (2005). Geneland: A computer package for landscape genetics. Molecular Ecology Notes, 5(3), 712–715. 10.1111/j.1471-8286.2005.01031.x

[ece36161-bib-0018] Heffelfinger, J. (2011). Taxonomy, evolutionary history, and distribution In Hewitt D. G. (Ed.), Biology and management of white‐tailed deer (1st ed., pp. 3–39). Boca Raton, FL: Taylor & Francis.

[ece36161-bib-0019] Hefley, T. J. , Hooten, M. B. , Russell, R. E. , Walsh, D. P. , & Powell, J. A. (2017). When mechanism matters: Bayesian forecasting using models of ecological diffusion. Ecology Letters, 20(5), 640–650. 10.1111/ele.12763 28371055

[ece36161-bib-0020] Holm, S. (1979). A simple sequentially rejective multiple test procedure. Scandinavian Journal of Statistics, 6(2), 65–70.

[ece36161-bib-0021] Homer, C. , Dewitz, J. , Yang, L. , Jin, S. , Danielson, P. , Coulston, J. , … Megown, K. (2015). Completion of the 2011 National Land Cover Database for the conterminous United States—Representing a decade of land cover change information. Photogrammetric Engineering and Remote Sensing, 81, 345–354.

[ece36161-bib-0022] Jombart, T. (2008). Adegenet: A R package for the multivariate analysis of genetic markers. Bioinformatics, 24(11), 1403–1405. 10.1093/bioinformatics/btn129 18397895

[ece36161-bib-0023] Kamvar, Z. N. , Tabima, J. F. , & Grünwald, N. J. (2014). Poppr: An R package for genetic analysis of populations with clonal, partially clonal, and/or sexual reproduction. PeerJ, 2, e281 10.7717/peerj.281 24688859 PMC3961149

[ece36161-bib-0024] Kelly, A. C. , Mateus‐Pinilla, N. E. , Brown, W. , Ruiz, M. O. , Douglas, M. R. , Douglas, M. E. , … Novakofski, J. (2014). Genetic assessment of environmental features that influence deer dispersal: Implications for prion‐infected populations. Population Ecology, 56(2), 327–340. 10.1007/s10144-013-0427-9

[ece36161-bib-0025] Ketchen, D. J. , & Shook, C. L. (1996). The application of cluster analysis in strategic management research: An analysis and critique. Strategic Management Journal, 17(6), 441–458. 10.1002/(SICI)1097-0266(199606)17:6<441:AID-SMJ819>3.0.CO;2-G

[ece36161-bib-0026] Langwig, K. E. , Voyles, J. , Wilber, M. Q. , Frick, W. F. , Murray, K. A. , Bolker, B. M. , … Kilpatrick, A. M. (2015). Context‐dependent conservation responses to emerging wildlife diseases. Frontiers in Ecology and the Environment, 13(4), 195–202. 10.1890/140241

[ece36161-bib-0027] Locher, A. , Scribner, K. T. , Moore, J. A. , Murphy, B. , & Kanefsky, J. (2015). Influence of landscape features on spatial genetic structure of white‐tailed deer in human‐altered landscapes. The Journal of Wildlife Management, 79(2), 180–194. 10.1002/jwmg.826

[ece36161-bib-0028] Long, E. S. , Diefenbach, D. R. , Rosenberry, C. S. , Wallingford, B. D. , & Grund, M. D. (2005). Forest cover influences dispersal distance of white‐tailed deer. Journal of Mammalogy, 86(3), 623–629. 10.1644/1545-1542(2005)86[623:FCIDDO]2.0.CO;2

[ece36161-bib-0029] Long, E. S. , Diefenbach, D. R. , Wallingford, B. D. , & Rosenberry, C. S. (2010). Influence of roads, rivers, and mountains on natal dispersal of white‐tailed deer. Journal of Wildlife Management, 74(6), 1242–1249. 10.2193/2009-096

[ece36161-bib-0030] Lutz, C. L. , Diefenbach, D. R. , & Rosenberry, C. S. (2015). Population density influences dispersal in female white‐tailed deer. Journal of Mammalogy, 96(3), 494–501. 10.1093/jmammal/gyv054

[ece36161-bib-0031] Lutz, C. L. , Diefenbach, D. R. , & Rosenberry, C. S. (2016). Proximate influences on female dispersal in white‐tailed deer. The Journal of Wildlife Management, 80(7), 1218–1226. 10.1002/jwmg.21106

[ece36161-bib-0032] Manjerovic, M. B. , Green, M. L. , Mateus‐Pinilla, N. , & Novakofski, J. (2014). The importance of localized culling in stabilizing chronic wasting disease prevalence in white‐tailed deer populations. Preventive Veterinary Medicine, 113(1), 139–145. 10.1016/j.prevetmed.2013.09.011 24128754

[ece36161-bib-0033] Mateus‐Pinilla, N. , Weng, H.‐Y. , Ruiz, M. O. , Shelton, P. , & Novakofski, J. (2013). Evaluation of a wild white‐tailed deer population management program for controlling chronic wasting disease in Illinois, 2003–2008. Preventive Veterinary Medicine, 110(3), 541–548. 10.1016/j.prevetmed.2013.03.002 23558033

[ece36161-bib-0034] McRae, B. H. , Dickson, B. G. , Keitt, T. H. , & Shah, V. B. (2008). Using circuit theory to model connectivity in ecology, evolution, and conservation. Ecology, 89(10), 2712–2724. 18959309 10.1890/07-1861.1

[ece36161-bib-0035] Meirmans, P. G. (2014). Nonconvergence in Bayesian estimation of migration rates. Molecular Ecology Resources, 14(4), 726–733. 10.1111/1755-0998.12216 24373147

[ece36161-bib-0036] Miller, M. W. , & Williams, E. S. (2004). Chronic wasting disease of cervids. Current Topics in Microbiology and Immunology, 284, 193–214. 15148993 10.1007/978-3-662-08441-0_8

[ece36161-bib-0037] Miller, W. L. , Edson, J. , Pietrandrea, P. , Miller‐Butterworth, C. , & Walter, W. D. (2019). Identification and evaluation of a core microsatellite panel for use in white‐tailed deer (*Odocoileus virginianus*). BMC Genetics, 20(1). 10.1186/s12863-019-0750-z PMC6554959 31170908

[ece36161-bib-0038] Miller, W. L. , & Walter, W. D. (In press). Can genetic assignment tests provide insight on the influence of captive egression on the epizootiology of chronic wasting disease? Evolutionary Applications. 10.1111/eva.12895 PMC7086050 32211062

[ece36161-bib-0039] Miller‐Butterworth, C. M. , Vonhof, M. J. , Rosenstern, J. , Turner, G. G. , & Russell, A. L. (2014). Genetic structure of little brown bats (*Myotis lucifugus*) corresponds with spread of white‐nose syndrome among hibernacula. Journal of Heredity, 105(3), 354–364. 10.1093/jhered/esu012 24591103

[ece36161-bib-0040] Nobert, B. R. , Merrill, E. H. , Pybus, M. J. , Bollinger, T. K. , & Hwang, Y. T. (2016). Landscape connectivity predicts chronic wasting disease risk in Canada. Journal of Applied Ecology, 53(5), 1450–1459. 10.1111/1365-2664.12677

[ece36161-bib-0041] Paquette, S. R. , Talbot, B. , Garant, D. , Mainguy, J. , & Pelletier, F. (2014). Modelling the dispersal of the two main hosts of the raccoon rabies variant in heterogeneous environments with landscape genetics. Evolutionary Applications, 7(7), 734–749. 10.1111/eva.12161 25469156 PMC4227855

[ece36161-bib-0042] Peakall, R. , & Smouse, P. E. (2006). GenAlEx 6: Genetic analysis in Excel. Population genetic software for teaching and research. Molecular Ecology Notes, 6(1), 288–295. 10.1111/j.1471-8286.2005.01155.x PMC3463245 22820204

[ece36161-bib-0043] Peakall, R. , & Smouse, P. E. (2012). GenAlEx 6.5: Genetic analysis in Excel. Population genetic software for teaching and research–an update. Bioinformatics, 28(19), 2537–2539. 10.1093/bioinformatics/bts460 22820204 PMC3463245

[ece36161-bib-0044] Pelletier, A. , Obbard, M. E. , Mills, K. , Howe, E. J. , Burrows, F. G. , White, B. N. , & Kyle, C. J. (2012). Delineating genetic groupings in continuously distributed species across largely homogeneous landscapes: A study of American black bears (*Ursus americanus*) in Ontario, Canada. Canadian Journal of Zoology, 90(8), 999–1014. 10.1139/z2012-068

[ece36161-bib-0045] Peterman, W. E. (2018). ResistanceGA: An R package for the optimization of resistance surfaces using genetic algorithms. Methods in Ecology and Evolution, 9(6), 1638–1647. 10.1111/2041-210X.12984

[ece36161-bib-0046] Robinson, S. J. , Samuel, M. D. , Lopez, D. L. , & Shelton, P. (2012). The walk is never random: Subtle landscape effects shape gene flow in a continuous white‐tailed deer population in the Midwestern United States. Molecular Ecology, 21(17), 4190–4205. 10.1111/j.1365-294X.2012.05681.x 22882236

[ece36161-bib-0047] Rosenberry, C. S. , & Diefenbach, D. R. (2019). Deer harvest variation in small and large management units in Pennsylvania. Wildlife Society Bulletin, 43(1), 71–76. 10.1002/wsb.939

[ece36161-bib-0048] Safner, T. , Miller, M. P. , McRae, B. H. , Fortin, M.‐J. , & Manel, S. (2011). Comparison of Bayesian clustering and edge detection methods for inferring boundaries in landscape genetics. International Journal of Molecular Sciences, 12(2), 865–889. 10.3390/ijms12020865 21541031 PMC3083678

[ece36161-bib-0049] Saunders, S. E. , Bartelt‐Hunt, S. L. , & Bartz, J. C. (2012). Occurrence, transmission, and zoonotic potential of chronic wasting disease. Emerging Infectious Diseases, 18(3), 369–376. 10.3201/eid1803.110685 22377159 PMC3309570

[ece36161-bib-0050] Scheele, B. C. , Pasmans, F. , Skerratt, L. F. , Berger, L. , Martel, A. N. , Beukema, W. , … Canessa, S. (2019). Amphibian fungal panzootic causes catastrophic and ongoing loss of biodiversity. Science, 363(6434), 1459–1463. 10.1126/science.aav0379 30923224

[ece36161-bib-0051] Sutherland, W. J. , Butchart, S. H. M. , Connor, B. , Culshaw, C. , Dicks, L. V. , Dinsdale, J. , … Gleave, R. A. (2018). A 2018 horizon scan of emerging issues for global conservation and biological diversity. Trends in Ecology & Evolution, 33(1), 47–58. 10.1016/j.tree.2017.11.006 29217396

[ece36161-bib-0052] Thogmartin, W. E. , Sanders‐Reed, C. A. , Szymanski, J. A. , McKann, P. C. , Pruitt, L. , King, R. A. , … Russell, R. E. (2013). White‐nose syndrome is likely to extirpate the endangered Indiana bat over large parts of its range. Biological Conservation, 160, 162–172. 10.1016/j.biocon.2013.01.010

[ece36161-bib-0053] Vergara, M. , Basto, M. P. , Madeira, M. J. , Gómez‐Moliner, B. J. , Santos‐Reis, M. , Fernandes, C. , & Ruiz‐González, A. (2015). Inferring population genetic structure in widely and continuously distributed carnivores: The Stone Marten (*Martes foina*) as a case study. PLoS ONE, 10, e0134257 10.1371/journal.pone.0134257 26222680 PMC4519273

[ece36161-bib-0054] Walter, W. D. , Baasch, D. M. , Hygnstrom, S. E. , Trindle, B. D. , Tyre, A. J. , Millspaugh, J. J. , … VerCauteren, K. C. (2011). Space use of sympatric deer in a riparian ecosystem in an area where chronic wasting disease is endemic. Wildlife Biology, 17(2), 191–209. 10.2981/10-055

[ece36161-bib-0055] Weir, B. S. , & Cockerham, C. C. (1984). Estimating F‐statistics for the analysis of population structure. Evolution, 38(6), 1358–1370. 10.2307/2408641 28563791

[ece36161-bib-0056] Wilson, G. A. , & Rannala, B. (2003). Bayesian inference of recent migration rates using multilocus genotypes. Genetics, 163(3), 1177–1191. 12663554 10.1093/genetics/163.3.1177 PMC1462502

