# Supplementary material for: Assessment of spatial genetic structure to identify populations at risk for infection of an emerging epizootic disease
Source: Ecol Evol. 2020 Apr 22;10(9):3977–90. doi: 10.1002/ece3.6161 (PMC7244803; doi:10.1002/ece3.6161)
Supplement: Supplementary file 1 — Figure S1 [file ECE3-10-3977-s001.docx]

#
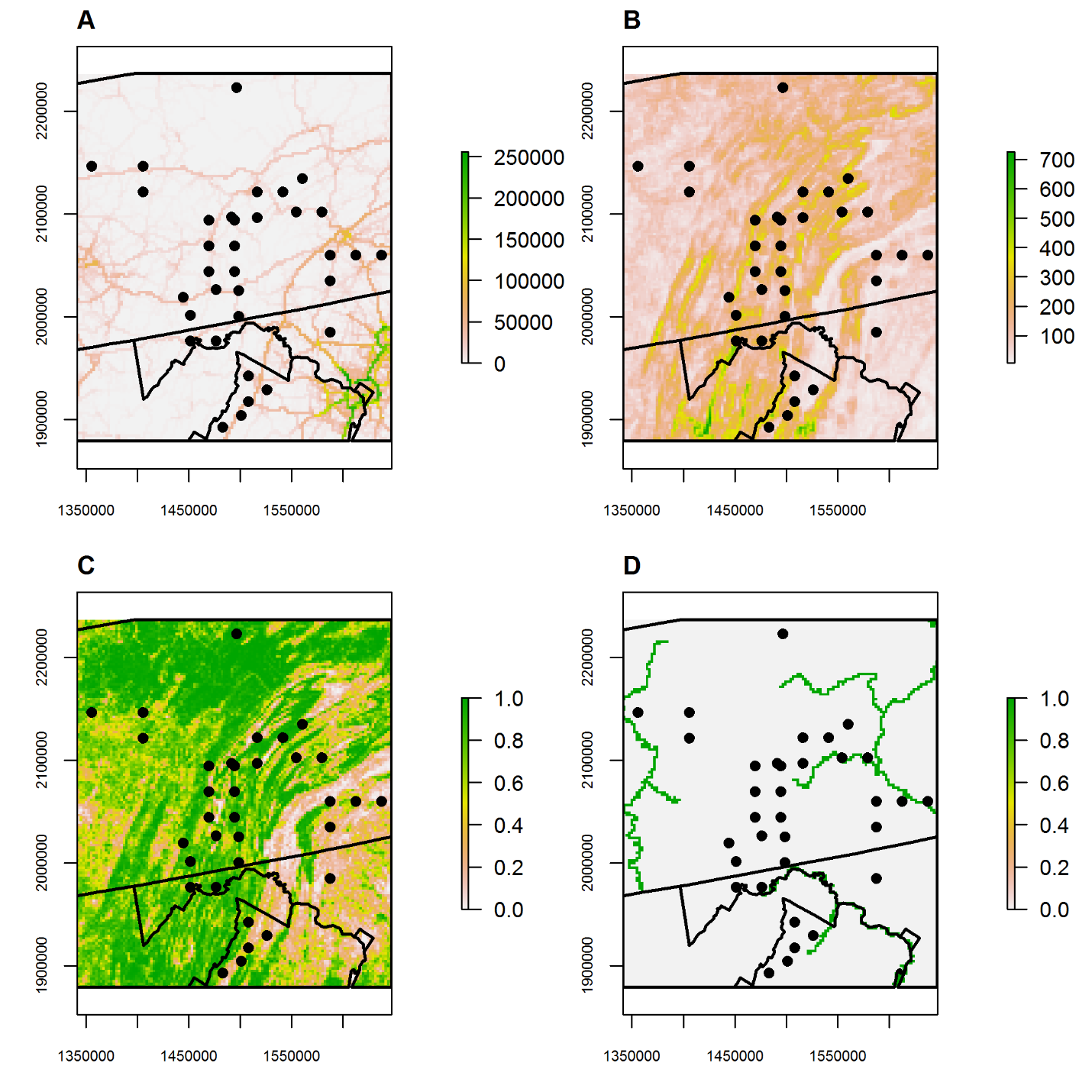


# Figure S1. Raster surfaces used to evaluate white-tailed deer genetic connectivity in the Mid-Atlantic region of the United States. (A) = average annual daily traffic volume, (B) = elevational relief, (C) percent forest cover, and (D) large streams (Strahler order ≥ 4). Black circles represent the centroid of 25 km x 25 km sampling grid cells used to subsample 11 genetic clusters identified in the Geneland analysis. Only sampling grids with ≥ 20 samples were included in landscape genetic analyses (number of grid cells = 34; total sample size = 1796).
