# Supplementary material for: Assessment of spatial genetic structure to identify populations at risk for infection of an emerging epizootic disease
Source: Ecol Evol. 2020 Apr 22;10(9):3977–90. doi: 10.1002/ece3.6161 (PMC7244803; doi:10.1002/ece3.6161)
Supplement: Supplementary file 2 — Table S1 [file ECE3-10-3977-s002.docx]

Table S1. Pairwise F_ST_ estimates (below diagonal) for 11 white-tailed deer subpopulations in the Mid-Atlantic region of the United States. Subpopulation identifiers correspond to designations outlined in Fig. 1. Significant deviations from panmixia are indicated above the diagonal with an asterisk (*P < 0.0009*).

|  | 1 | 2 | 3 | 4 | 5 | 6 | 7 | 8 | 9 | 10 | 11 |
| --- | --- | --- | --- | --- | --- | --- | --- | --- | --- | --- | --- |
| 1 | 0 | * | * | * | * | * | * | * | * | * | * |
| 2 | 0.010 | 0 | * | * | * | * | * | * | * | * | * |
| 3 | 0.015 | 0.015 | 0 | * | * | * | * | * | * | * | * |
| 4 | 0.026 | 0.025 | 0.008 | 0 | * | * | * | * | * | * | * |
| 5 | 0.020 | 0.014 | 0.013 | 0.024 | 0 | * | * | * | * | * | * |
| 6 | 0.020 | 0.015 | 0.010 | 0.007 | 0.011 | 0 | * | * | * | * | * |
| 7 | 0.026 | 0.019 | 0.018 | 0.023 | 0.007 | 0.009 | 0 | * | * | * | * |
| 8 | 0.022 | 0.013 | 0.013 | 0.019 | 0.009 | 0.006 | 0.004 | 0 | * | * | * |
| 9 | 0.021 | 0.017 | 0.021 | 0.027 | 0.015 | 0.017 | 0.014 | 0.015 | 0 | * | * |
| 10 | 0.030 | 0.023 | 0.023 | 0.033 | 0.023 | 0.022 | 0.017 | 0.015 | 0.011 | 0 | * |
| 11 | 0.023 | 0.022 | 0.028 | 0.041 | 0.024 | 0.033 | 0.030 | 0.029 | 0.022 | 0.033 | 0 |
