# Supplementary material for: Assessment of spatial genetic structure to identify populations at risk for infection of an emerging epizootic disease
Source: Ecol Evol. 2020 Apr 22;10(9):3977–90. doi: 10.1002/ece3.6161 (PMC7244803; doi:10.1002/ece3.6161)
Supplement: Supplementary file 3 — Table S2 [file ECE3-10-3977-s003.docx]

Table S2. Unidirectional migration rates for 11 white-tailed deer subpopulations in the Mid-Atlantic region of the United States estimated using BayesAss. Column headers represent the source subpopulation and the row headers represent the recipient subpopulation. The proportion of resident individuals within each subpopulation is displayed on the diagonal (italicized). Bold values indicate migration estimates where 95% credible intervals did not bound zero.

|  | 1 | 2 | 3 | 4 | 5 | 6 | 7 | 8 | 9 | 10 | 11 |
| --- | --- | --- | --- | --- | --- | --- | --- | --- | --- | --- | --- |
| 1 | ***0.936*** | **0.033** | **0.002** | **0.011** | 0.002 | **0.005** | 0.002 | **0.002** | 0.002 | **0.003** | 0.003 |
| 2 | **0.014** | ***0.941*** | 0.002 | **0.004** | **0.003** | **0.014** | **0.005** | **0.004** | **0.005** | **0.005** | **0.005** |
| 3 | **0.013** | **0.029** | ***0.674*** | **0.201** | **0.010** | **0.019** | **0.019** | **0.012** | **0.007** | **0.009** | **0.008** |
| 4 | **0.004** | **0.003** | 0.002 | ***0.856*** | **0.002** | **0.110** | **0.006** | 0.002 | **0.003** | **0.006** | **0.006** |
| 5 | **0.014** | **0.018** | **0.005** | **0.015** | ***0.706*** | **0.015** | **0.188** | **0.008** | **0.007** | **0.010** | **0.015** |
| 6 | **0.003** | **0.035** | 0.002 | **0.030** | **0.006** | ***0.838*** | **0.064** | **0.015** | **0.004** | **0.002** | **0.002** |
| 7 | **0.005** | **0.006** | 0.004 | **0.006** | **0.006** | **0.059** | ***0.863*** | **0.010** | **0.025** | **0.009** | **0.007** |
| 8 | **0.011** | **0.019** | **0.003** | **0.004** | **0.004** | **0.063** | **0.192** | ***0.680*** | **0.008** | **0.014** | **0.004** |
| 9 | **0.012** | **0.012** | 0.003 | **0.009** | **0.010** | **0.011** | **0.028** | **0.004** | ***0.783*** | **0.112** | **0.016** |
| 10 | **0.007** | **0.011** | 0.003 | **0.004** | **0.004** | **0.006** | **0.045** | 0.004 | **0.030** | ***0.880*** | **0.005** |
| 11 | **0.004** | **0.006** | 0.002 | **0.002** | **0.003** | **0.003** | **0.006** | **0.002** | **0.004** | **0.002** | ***0.965*** |
