# Supplementary material for: Assessment of spatial genetic structure to identify populations at risk for infection of an emerging epizootic disease
Source: Ecol Evol. 2020 Apr 22;10(9):3977–90. doi: 10.1002/ece3.6161 (PMC7244803; doi:10.1002/ece3.6161)
Supplement: Supplementary file 4 — Appendix S1 [file ECE3-10-3977-s004.docx]

# Appendix A: Description and results of quality control procedures for microsatellite genotyping of white-tailed deer populations sampled from the Mid-Atlantic region of the United States.

**Materials and Methods**

We reanalyzed a total of 225 samples (approximately 10.1% of the data) to ensure reproducibility of results. Genotyping error rates were calculated for the entire dataset and per locus and were defined as the number of mismatched alleles divided by the total number of alleles. For null allele estimates and evaluation of equilibrium assumptions, samples were grouped by county, a courser spatial resolution than the initial sampling scheme, in order to increase sample size by locality and increase statistical power. We estimated the frequency of null alleles in the dataset using the FreeNA algorithm (Chapuis & Estoup, 2007). Null alleles were estimated in the total dataset and for each free-ranging population with a sample size ≥ 25 individuals. Corrected F_ST_ values were compared to uncorrected F_ST_ values to determine the influence of observed null alleles on estimates of population structure. The program Genepop (version 4.6; Raymond & Rousset, 1995; Rousset, 2008) was used to evaluate deviations from Hardy-Weinberg and Linkage expectations using default Markov Chain Monte Carlo parameters (10,000 dememorizations, 1000 batches, 10,000 iterations per batch). Significance was assessed using the Holm-Bonferroni procedure for multiple comparisons (Holm 1979).

**Results**

We were successfully able to genotype all individuals across 11 loci in >99% of cases. No individuals were missing genotypes at more than two loci. The estimated genotyping error rate for the entire dataset was 0.29%. The per locus error rate did not exceed 0.44% for any locus. Null allele frequencies did not exceed 5.9% per locus with an average of 2.9% among loci. Significant null allele frequencies (>10% frequency) were detected in three locus by county comparisons (out of 253) and never exceeded 12.4%. There was strong collinearity between F_ST_ estimates uncorrected for null alleles and those that were corrected (*r* >0.99, maximum difference = 0.002, mean difference < 0.001), indicating that the observed frequency of null alleles did not produce an appreciable effect on population genetics estimates. Deviations from Hardy-Weinberg expectations occurred in 4.0% of locus by county comparisons, with no single locus exceeding deviations in > four out of 23 counties. No loci were found to be in linkage disequilibrium across counties. These results suggest that genotyping errors were rare and not expected to significantly influence estimates of genetic structure and/or connectivity, so we elected to include all individuals and loci in subsequent analyses.

**References**

Chapuis, M.-P., & Estoup, A. (2007). Microsatellite null alleles and estimation of population differentiation. *Molecular Biology and Evolution*, *24*(3), 621–631. doi:10.1093/molbev/msl191

Raymond, M., & Rousset, F. (1995). GENEPOP (version 1.2): Population genetics software for exact tests and ecumenicism. *Journal of Heredity*, *86*(3), 248–249.

Rousset, F. (2008). Genepop’007: A complete re-implementation of the genepop software for Windows and Linux. *Molecular Ecology Resources*, *8*(1), 103–106. doi:10.1111/j.1471-8286.2007.01931.x

Holm S. 1979. A simple sequentially rejective multiple test procedure. *Scandinavian Journal of Statistics,* *6*, 65–70.
