# Supplementary material for: Assessment of spatial genetic structure to identify populations at risk for infection of an emerging epizootic disease
Source: Ecol Evol. 2020 Apr 22;10(9):3977–90. doi: 10.1002/ece3.6161 (PMC7244803; doi:10.1002/ece3.6161)
Supplement: Supplementary file 5 — Appendix S2 [file ECE3-10-3977-s005.docx]

# Appendix B. Comparison of the output from the Geneland algorithm for the two Markov Chain Monte Carlo runs with the highest log-likelihoods.


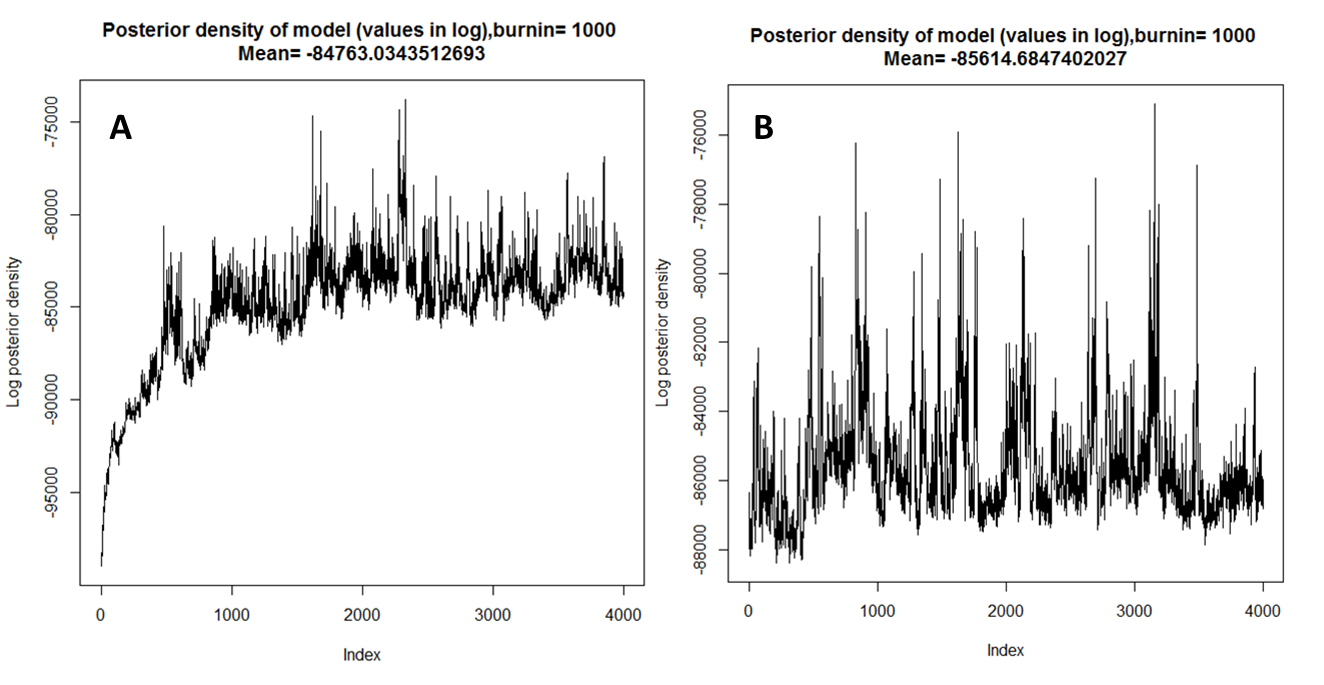


**Figure B1.** Trace plots for the Geneland analyses with (A) the highest log posterior density and (B) the second highest log posterior density following a burn-in period of 1000 iterations.


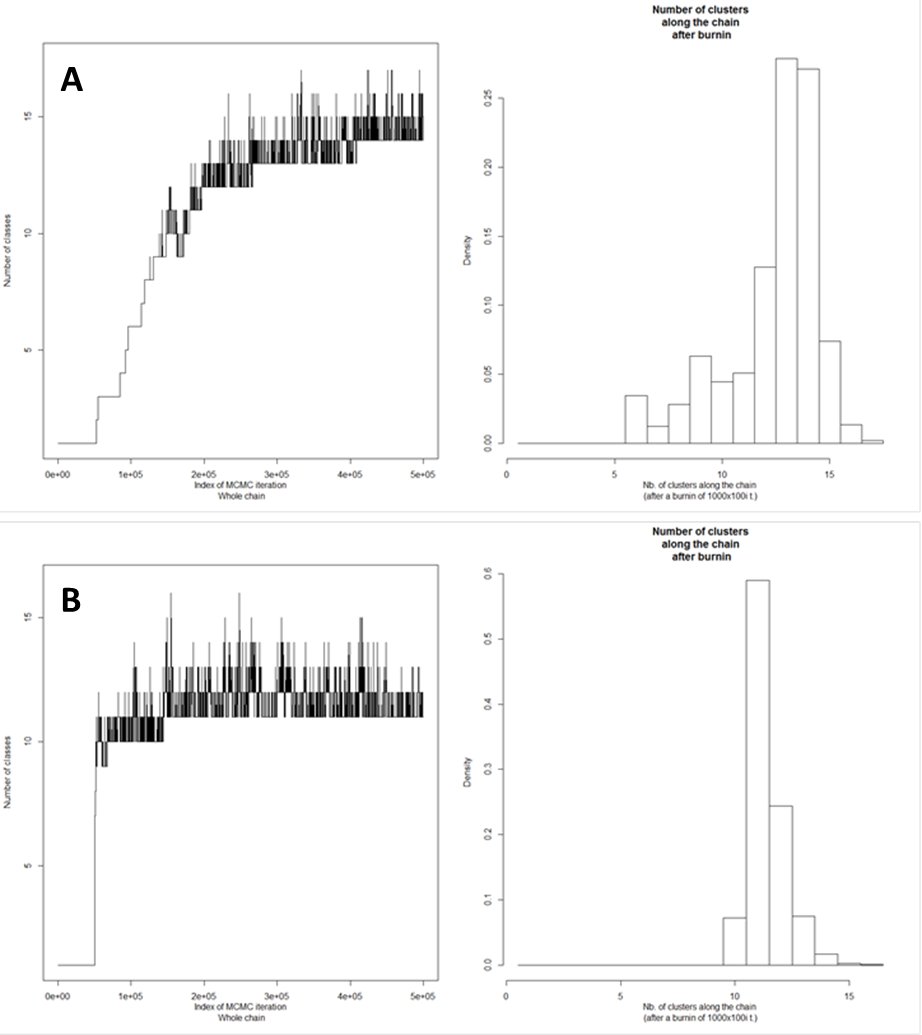


**Figure B2.** Plots of the number of genetic clusters (*K*) over all iterations (left) and the total density of *K* along the chain following a burn-in period of 1000 iterations (right) for the MCMC chains with (A) the highest log posterior density and (B) the second highest log posterior density.
